# A303 SAFETY AND EFFICACY OF DIRECT ORAL ANTICOAGULANTS IN PATIENTS WITH LIVER CIRRHOSIS: A SYSTEMATIC REVIEW AND META-ANALYSIS

**DOI:** 10.1093/jcag/gwad061.303

**Published:** 2024-02-14

**Authors:** D Tham, W Yu, L Zhao, R Kou, J Kherani, P Li, S Sreeraman, A Eshaghpour, A Li, M Crowther

**Affiliations:** University of Ottawa Faculty of Medicine, Ottawa, ON, Canada; University of Ottawa Faculty of Medicine, Ottawa, ON, Canada; McMaster University Faculty of Health Sciences, Hamilton, ON, Canada; University of Ottawa Faculty of Medicine, Ottawa, ON, Canada; McMaster University Faculty of Health Sciences, Hamilton, ON, Canada; University of Toronto Temerty Faculty of Medicine, Toronto, ON, Canada; Western University Schulich School of Medicine & Dentistry, London, ON, Canada; McMaster University Department of Medicine, Hamilton, ON, Canada; University of Ottawa Faculty of Medicine, Ottawa, ON, Canada; McMaster University Department of Medicine, Hamilton, ON, Canada

## Abstract

**Background:**

Direct oral anticoagulants (DOACs) are widely used for treatment of venous thromboembolism (VTE) and stroke prophylaxis in patients with atrial fibrillation. Liver cirrhosis increases the risk of conditions necessitating anticoagulation, but also increases bleeding risks, complicating anticoagulation use. DOACs may be suitable for chirrotic patients as their reliance on hepatic elimination is reduced compared to traditionally used anticoagulants such as low molecular weight heparin (LMWH) or vitamin K antagonists (VKAs). However, safety data on DOACs in cirrhosis patients is limited as severe Child-Pugh (CP) classes are historically underrepresented in major trials. Various guidelines currently classify severe CP classes as contraindications to DOACs.

**Aims:**

To synthesize primary evidence on the safety profile of DOACs in patients with severe liver cirrhosis.

**Methods:**

A literature search of MEDLINE and Embase from inception to Jan 2023 identified randomized controlled trials (RCTs) and cohort studies comparing DOACs to LMWH or VKAs in cirrhosis patients. Two reviewers screened and extracted data at title/abstract, and full-text levels. Baseline patient characteristics, CP class, and anticoagulation regimens were extracted. The primary outcome was major bleeding per ISTH criteria, stratified by CP B&C, and CP Unspecified (primary study did not report/stratify by CP class) subgroups. Data were meta-analyzed using the Mantel-Haenszel random-effects model and presented as odds ratios with corresponding 95% confidence intervals.

**Results:**

Of 794 articles screened, 17 articles (2 RCTs, 15 cohorts) were included for analysis (n = 5046). Overall, DOACs were associated with a lower risk of major bleeding compared to controls (OR=0.63 [0.45, 0.89]). This result was consistent in the CP B&C Exclusive subgroup (OR=0.42 [0.29, 0.62], 5 studies) but not in the CP Unspecified subgroup (OR=0.71 [0.48, 1.08], 12 studies). One large study (n=3213), Lawal 2023, in the CP B&C group, favored DOACs in reduction of major bleeding (OR=0.37, 95%CI [0.24,0.57]). Removal of this study in post-hoc sensitivity analysis led to no significant differences in major bleeding overall or in any subgroup.

**Conclusions:**

In this analysis comparing DOACs to LMWH/VKAs in patients with liver cirrhosis, DOACs were associated with a statistically significant reduction in the risk of major bleeding overall and in more advanced subgroups (CP B&C). Future analysis will stratify by DOAC agent, duration, dosage, and include secondary efficacy outcomes.

Table 1. Summary of the major bleeding effect size in all subgroups.

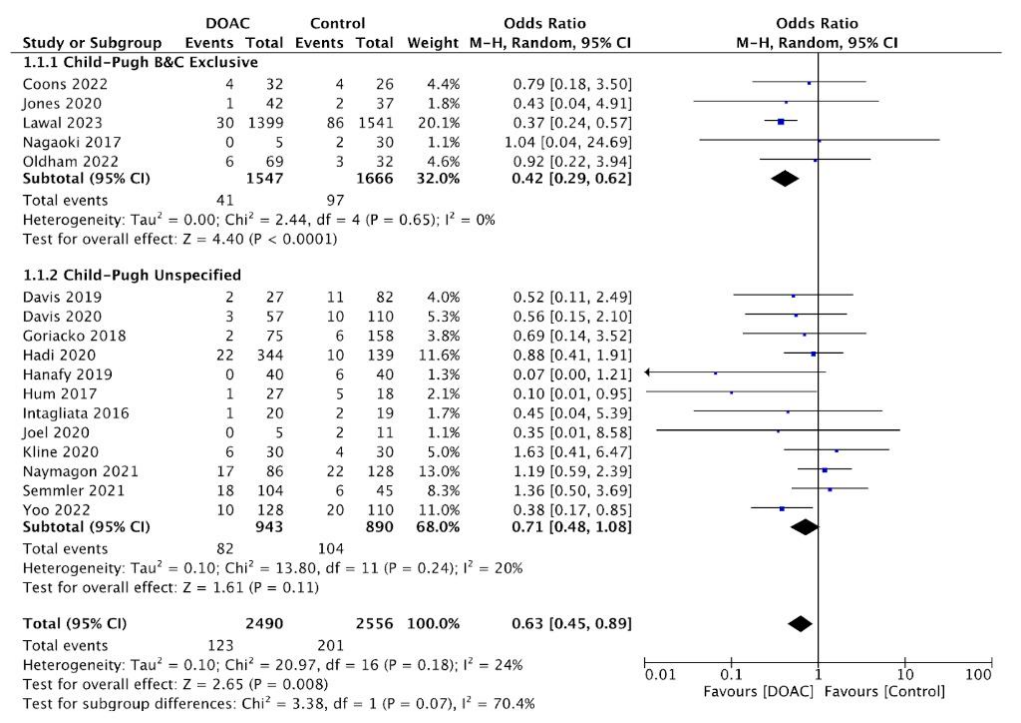

Figure 1. Major bleeding in Child-Pugh B&C and Child-Pugh Unspecified groups with DOAC vs VKA

**Funding Agencies:**

None

**Table:** 

Subgroup (Major Bleeding)	DOAC’s (events/total sample)	LMWH/VKA (events/total sample)	Effect Size	Number of Studies in Meta-Analysis
CP Class B&C	41/1547	97/1666	OR = 0.42 (95% CI: 0.29 to 0.62; p=ampersand:003C0.0001; I2=0%)	5
CP Unspecified	82/943	104/890	OR = 0.71 (95% CI: 0.48 to 1.08; p=0.11; I2=20%)	12
Overall	123/2490	201/2556	OR = 0.63 (95% CI: 0.45 to 0.89; p=0.008; I2=24%)	17

